# Dual sourcing inventory management with nonconsecutive lead times from a supply chain perspective: a numerical study

**DOI:** 10.1007/s00291-023-00720-4

**Published:** 2023-05-10

**Authors:** Younes Hamdouch, Youssef Boulaksil, Kilani Ghoudi

**Affiliations:** 1 College of Business, Zayed University, Dubai, United Arab Emirates; 2 College of Business and Economics, UAE University, Al Ain, United Arab Emirates

**Keywords:** Inventory management, Dual sourcing, Nonconsecutive lead times, Supply chain, TBS policy, DIP policy, Policy evaluation

## Abstract

We study a stochastic multi-period two-echelon dual sourcing inventory system where the buyer can source a product from two different suppliers: a regular and an expedited supplier. The regular supplier is a low-cost offshore supplier, whereas the expedited supplier is a responsive nearshore supplier. Such dual sourcing inventory systems have been well studied in the literature, mostly being solely evaluated from the buyer’s perspective. Since the buyer’s decisions have an impact on the supply chain profit, we adopt the perspective of the entire supply chain, i.e., by taking the suppliers explicitly into consideration. In addition, we study this system for *general (nonconsecutive) lead times* for which the optimal policy is unknown or very complex. We numerically compare the performance of two different policies in a two-echelon setting: the Dual-Index Policy (DIP) and the Tailored Base-Surge Policy (TBS). From earlier studies we know that when the lead time difference is one period, DIP is optimal from the buyer’s perspective, but not necessarily from the supply chain perspective. On the other hand, when the lead time difference grows to infinity, TBS becomes optimal for the buyer. In this paper, we evaluate the policies numerically (under various conditions) and we show that from *a supply chain perspective*, TBS typically outperforms DIP at a limited lead time difference of a few time periods. Based on data collected from 51 manufacturing firms, the results of our paper imply for many supply chains with a dual sourcing setting that TBS quickly becomes a beneficial policy alternative, especially given its simple and appealing structure.

## Introduction

Supply chains are often complex networks of several suppliers and manufacturing facilities, mostly spread all over the world. Due to the increasing importance of offering a high service level in several industries, especially after the COVID-19 crisis has shown that supply chains are very vulnerable (Chowdhury et al. 2021), many firms aim for having a second (onshore) supplier with a short lead time in addition to the offshore supplier (Heckmann et al. 2015), which improves the responsiveness to customers (Snyder et al. 2016). Having two suppliers (or two different transportation modes) for the same product with a low-cost offshore supplier (regular supplier) and a responsive on-shore (expedited/emergency) supplier is what we refer to as a *dual sourcing* system, which is nowadays increasingly adopted in many supply chains (Busellato et al. 2021). Such a system combines two main advantages: *cost efficiency* by utilizing the regular supplier and increased *responsiveness* and *flexibility* by utilizing the expedited supplier (Yang et al. 2022). The trade-off between utilizing the two different suppliers is one of the main motivations for this study.

Several studies have shown the analysis of a dual sourcing strategy compared to a single sourcing strategy (Geetha and Achary 2000; Inderst 2008; Mukherjee and Sarin 2018; Knofius et al. 2021). The reported benefits are often in terms of cost savings (Tagaras and Vlachos 2001; Johansen and Thorstenson 1998) and improved bargaining position (Du et al. 2006). In addition, outsourcing, offshoring, and the increased availability of different transportation modes also contributed to the popularity of dual sourcing strategies in many industries and supply chains. Many studies have reported the benefits of successful implementations of dual sourcing strategies (Rao et al. 2000; Beyer and Ward 2002; Allon and Van Mieghem 2010). In an empirical study that we conducted among 51 manufacturing firms, we also found that almost all of them have adopted a dual- or multi-sourcing strategy for the supply of critical products for their production process.

In opposite to most single sourcing inventory systems, optimal policies for most dual sourcing inventory systems are unknown or have a very complex structure (Whittemore and Saunders 1977; Zipkin 2000). Despite the high relevance of dual sourcing strategies for many firms, finding the optimal policy for the general dual sourcing system, especially in case of nonconsecutive lead times is extremely difficult. Therefore, several simpler (heuristic) policies have been proposed in the literature, such as the Dual-Index Policy (DIP) (Veeraraghavan and Scheller-Wolf 2008) and the Tailored Base Surge Policy (TBS) (Janssen and De Kok 1999; Allon and Van Mieghem 2010).

The TBS policy combines a push and pull approach by ordering every period a constant quantity from the regular supplier (push approach) and ordering from the expedited supplier in a critical situation (pull approach). An advantage of this policy is that the regular supplier would benefit from constant order quantities and it is therefore appealing to implement in a real-life situation. On the other hand, DIP adopts solely a pull approach and suggests two basestock levels (control parameters), one for each of the two suppliers. In terms of the performance of these two policies, we know that for a single-echelon system, i.e., from the buyer’s perspective, the TBS policy has been proven to be asymptotically optimal (Xin and Goldberg 2018). That is, TBS is optimal when the lead time difference between the two suppliers grows to infinity. On the other hand, for the buyer, DIP has been proven to be optimal for consecutive the lead time differences, that is, when the lead time difference between the two suppliers is one time period (Fukuda 1964). Although we know what is optimal for the buyer, the performance of these two policies *from a supply chain perspective* for *general (nonconsecutive) lead times* remains unclear, which is the topic of this paper.

In this paper, we study a single-product, two-echelon, multi-period, periodic-review dual sourcing inventory system with general lead time differences between the two suppliers, given that the buyer faces uncertain external demand. We compare the performance of two policies (DIP and TBS), while explicitly studying the impact of the buyer’s decisions on the suppliers and on the entire supply chain. This perspective has been proven to give different insights that are not obtained when only studying a single-echelon setting (Boulaksil et al. 2021). For example, when the lead time difference between the suppliers is one time period (consecutive lead times), DIP is optimal for the buyer, but not necessarily for the entire supply chain.

One of the main result of this paper is that in a supply chain setting, TBS mostly outperforms DIP after a lead time difference of a few time periods, which may not be intuitive. The DIP policy employs two basestock levels, which means that the order quantities to both suppliers can be varied at any time period, which TBS cannot. Therefore, one may expect that this lack of flexibility puts TBS at a disadvantage, which appears to be not the case when the lead time difference grows. On the contrary, for many realistic dual sourcing settings, TBS becomes an interesting and recommended policy alternative when the lead time difference is more than a few time periods, especially given its simple and appealing structure. This has been confirmed by the data that we collected from 51 manufacturing companies that have suppliers located all over the world. The collected data are used for our extensive numerical study in which several model parameters have been varied. We present insights about the break-even lead time difference and we show that the performance of these companies can be substantially improved if the insights from this study are implemented. The results of our study may have serious implications and are of interest to practitioners that deal with dual sourcing supply chains.

The contribution of this study is threefold. First, we study the dual sourcing problem for the general lead times case from a supply chain perspective by explicitly taking the impact of the buyer’s decisions on the suppliers into consideration. Based on an extensive literature review, Svoboda et al. (2021) concluded that in the dual sourcing inventory management literature, multi-echelon studies are still largely an open field. Moreover, the vast majority of studies in this field assumes a lead time difference of one time period, which is often an unrealistic scenario. For example, a recent study in the e-commerce business shows that many products sold at Walmart.com that have two suppliers (a local and an overseas one) face a lead time difference of up to 12 weeks (Xin et al. 2017). Second, we present analytical results that show the effects of several model parameters on the profit functions. In addition, our numerical study reveals insights on the break-even lead time difference that determines the preferred policy from a supply chain perspective. Third, we collect data from 51 manufacturing companies for model validation purposes and to confirm the added value of our work for practitioners.

The remainder of this paper is organized as follows. In Sect. 2, we review the relevant literature. Then, in Sect. 3, the model formulation and an analysis of both policies are presented. In Sect. 4, we provide some analytical results to illustrate the effects of the model parameters on the expected profits and present the results of our numerical study, and in Sect. 5, we present the main results of the collected data along with a discussion of some managerial insights. Finally, in Sect. 6, the main conclusions from this study as well as ideas for future research are discussed.

## Literature review

The dual sourcing inventory management problem has been well studied and received an increasing attention in the last two decades (Svoboda et al. 2021). Below, we discuss the most relevant streams in the literature with a focus on studies that consider general lead times in a periodic-review setting. At the end of the section, we discuss our main contributions to the literature. For a more extensive literature review on this topic, we refer the reader to the following two excellent review papers: Minner (2003) and Svoboda et al. (2021).

Barankin (1961) was the first to study a dual sourcing inventory management problem. That study considered a single-period model with a regular supply after one time period in addition to an immediate expedited supply (with zero lead time). The author found that the optimal policy structure consists of two basestock levels. Fukuda (1964) extended that study and derived the optimal policy for consecutive lead times, which is the setting where the lead time difference is one time period, independent of the lead time of the regular supplier. The author proved that a dual-basestock policy is then optimal, which also specifies two basestock levels, one for each supplier. This policy which tracks two basestock levels against two inventory positions has later been called the Dual-Index Policy (DIP) (Veeraraghavan and Scheller-Wolf 2008). In case of a lead time difference of one time period, this policy is optimal for the buyer, but not necessarily optimal for the entire supply chain (Boulaksil et al. 2021).

When the lead time difference becomes larger than one time period (nonconsecutive lead times), which is the setting we are studying in this paper, Whittemore and Saunders (1977) found that the optimal policy for the buyer (single-echelon system) has a complex structure. Basically, when the lead time difference grows, the optimal order decisions become dependent on all outstanding orders during the lead time difference, and therefore, the optimal policy is not anymore a function of one or two inventory positions. Consequently, in order to determine the optimal policy parameters, a multi-dimensional modeling approach is required, which becomes easily intractable due to the curse of dimensionality.

By proving that single-sourcing lost-sales inventory models with a positive lead time are a special case of the dual sourcing problem, Sheopuri et al. (2010) found that basestock policies are generally not optimal when the lead time difference between the supplier is larger than one and they proposed several better performing policies that make better use of the pipeline inventory information. The relationship with lost-sales inventory models is that the lost sales in a certain period is what should be ordered from the expedited supplier. Lost-sales inventory problems with lead times are very challenging problems (Karlin and Scarf 1958; Zipkin 2008; Huh et al. 2009). Several studies followed that looked specifically into the outstanding orders during the lead time difference. For example, Li and Yu (2014) characterized the monotonicity of the order quantities related to the outstanding orders during the lead time difference. Hua et al. (2015) showed that the optimal order quantity to the regular supplier is more sensitive to the longest outstanding orders, while the optimal order to the expedited supplier is more sensitive to the soon-to-arrive outstanding orders.

To summarize, when the lead time difference grows, optimal policies do not have a simple structure like basestock policies. In fact, the optimal order quantities become complex functions of a state vector with a length equal to the lead time difference between the two suppliers, which makes it hard to obtain optimal policies. Therefore, most studies in the literature focus on developing heuristic policies, i.e., good performing policies with a simpler structure. Below, we will review two well-studied (heuristic) policies in the literature: the Dual-Index Policy (DIP) and the Tailored Base-Surge (TBS) policy, which we will both use in this study.

### Dual-index policy

Veeraraghavan and Scheller-Wolf (2008) introduced the name Dual-Index Policy (DIP) for a dual sourcing inventory problem. DIP policy keeps track of two inventory positions: regular and expedited inventory positions and it consists of two basestock levels, one for each of the two suppliers. The policy works as follows. If the expedited inventory position (on-hand inventory level plus outstanding orders that will arrive within the emergency lead time minus the backorders) is less than the lower basestock level at a periodic review moment, then the difference is ordered from the expedited supplier. Then, the regular inventory position is raised to the higher basestock level by ordering from the regular supplier. In Veeraraghavan and Scheller-Wolf (2008), the two basestock levels are computed by using a simulation-based optimisation approach. The results of their numerical study show that the performance of DIP is close to optimal. Feng et al. (2006) found that in case the number of suppliers is more than two, only the fastest two suppliers have optimal basestock levels. As discussed before, Sheopuri et al. (2010) found that basestock policies are generally not optimal in case of nonconsecutive lead times. This inspired them to develop two groups of policies. One group of policies has a basestock level structure for the expedited supplier and the other one has a basestock level structure for the regular supplier. The developed policies (vector base-stock, weighted dual index, and demand allocation policies) perform very well and sometimes outperform DIP. Fong (1992), Song and Zipkin (2009), and Arts et al. (2011) all studied DIP by considering stochastic lead times. Arts and Kiesmüller (2013) develop a Markovian formulation of DIP for a two-stage system where the upstream stockpoint has two supply options. Recently, Sun and Van Mieghem (2019) extended DIP by proposing an upper limit (cap) on the orders to the regular supplier. They proved the (robust) optimality of this policy and they show that this policy increasingly smooths orders to the regular supplier as the lead time difference between the two suppliers grows. Basically, this means that the proposed policy converges to the TBS policy.

### Tailored base-surge policy

The second relevant stream of papers in the literature is the papers that study the TBS policy. The TBS policy is a special case of the so-called Standing Order Policies (SOP). Under SOP, the regular supplier delivers each period a constant quantity, which is also referred to as the standing order quantity, while the expedited supplier is controlled by a more flexible policy. It is a particular example of a single-index policy that combines a push approach (supply of a fixed quantity from the regular supplier) with a pull approach (ordering variable quantities from the expedited supplier). From a practical perspective, it is an attractive policy to implement, especially for the regular supplier who can operate very efficiently. On the other hand, the expedited supplier plays a more important role for the buyer to maintain a high service level.

Rosenshine and Obee (1976) were the first to study SOP. Their policy suggests that when the inventory position drops below a critical level, the expedited supplier will deliver with zero lead time, such that the inventory position is raised to a basestock level. Janssen and De Kok (1999) analyze a similar policy and optimise the policy parameters such that costs are minimized given a certain service level constraint. That study also showed that the level of demand uncertainty is the main factor that determines the optimal standing order quantity. Chiang (2007) also derived the optimal parameters for this policy, but given a standing order quantity that is determined exogenously. Allon and Van Mieghem (2010) introduced the name of Tailored Base-Surge (TBS) policy. It is a special case of SOP where the expedited supplier is controlled via a basestock policy. The authors present a simple square root formula to determine the near-optimal standing order quantity. Janakiraman et al. (2014) built on this and showed numerically that the performance difference between the optimal policy and TBS decreases as the lead time of the regular supplier increases. Recently, Xin and Goldberg (2018) proved that the TBS policy is asymptotically optimal, that is, when the lead time difference between the two suppliers grows to infinity. Xin et al. (2017) extended the proof of the asymptotic optimality of the TBS policy by considering random capacities at the expedited supplier.

### Policy comparison

From the literature, we learn that exact and optimal solutions are usually not achieved for the DIP policy under the general lead time case. In addition, it is only optimal when the lead time difference is one time period. On the other hand, we learn that TBS is asymptotically optimal. In this paper, we compare those two policies for the general lead time case. The two policies have been compared in three earlier studies. Klosterhalfen et al. (2011) and Sun and Van Mieghem (2019) compare TBS with DIP in a single-echelon setting and find that TBS sometimes outperforms DIP, especially when the level of demand uncertainty is high and the lead time difference is large. Boulaksil et al. (2021) also compare the performance of TBS with DIP in a two-echelon setting, but assuming a lead time difference of one time period. A recent study of Gijsbrechts et al. (2021) studied the possibility of using a deep reinforcement learning approach to find a good performing policy for a dual sourcing system. They show that their approach performs well compared to other policies.

Our main contribution to the literature is that, in opposite to the vast majority of the literature, we study the stochastic dual sourcing inventory management problem from a *supply chain perspective*, assuming *nonconsecutive lead times*. Despite that it has been proven that TBS is asymptotically optimal for the buyer, we find that in a supply chain setting, TBS outperforms DIP when the lead time difference is only a limited number of periods. Our collected data from 51 manufacturing firms confirms that this is a very realistic setting, which implies that TBS can be an appealing policy alterative for many supply chains that face a dual sourcing setting.

## Model formulation

In this section, we first present the main model assumptions. Then, we present the mathematical formulation of the buyer and the two suppliers’ models under both policies.

### Model assumptions

All used symbols are defined below:

**Table:** 

*t*	Period index ($1\le t\le T$)
$l_e$	Lead time from the expedited supplier to the buyer
$l_r$	Lead time from the regular supplier to the buyer
$\Delta l$	The lead time difference between the two suppliers $(= l_r-l_e)$
$Q_{t}^{e}$	Order quantity from the expedited supplier in period *t* under DIP policy
$Q_{t}^{r}$	Order quantity from the regular supplier in period *t* under DIP policy
$\widehat{Q}_{t}^{e}$	Order quantity from the expedited supplier under TBS policy
$\widehat{Q}^{r}$	Standing order quantity from the regular supplier under TBS policy
$Y^e$	Expedited basestock level under DIP policy
$Y^r$	Regular basestock level under DIP policy
$\Delta$	The difference between the two basestock levels $(\Delta = Y^r -Y^e$)
$\widehat{Y}$	Basestock level under TBS policy
$D_{t}$	Stochastic (external) demand faced by the buyer in period *t*
$\mu$	Mean (external) demand
$CV(D_t)$	Coefficient of variation of $D_t$
$I_{t}$	On-hand inventory level of the buyer at the beginning of period *t*
$IP_{t}^e$	Expedited inventory position in period *t* under DIP policy
$IP_{t}^r$	Regular inventory position in period *t* under DIP policy
$\widehat{IP}_{t}^{e}$	Expedited inventory position in period *t* under TBS policy
*h*	Unit inventory holding cost of the buyer
*b*	Unit backorder (penalty) cost of the buyer
*p*	Unit selling price of the buyer
$w_e$, $w_r$	Unit wholesale cost of the buyer from the expedited (e) and regular (r) suppliers
$\Delta w$	The wholesale price difference between the two suppliers $(\Delta w=w_e-w_r)$
$c_e$, $c_r$	Unit manufacturing (or purchasing) cost of the expedited (e) and regular (r) suppliers
$\Delta c$	The manufacturing (purchasing) difference between the two suppliers $(\Delta c=c_e-c_r)$
$\pi ^B$, $\pi ^{e}$, $\pi ^{r}$, $\pi$	Expected profit of the buyer, expedited and regular suppliers, total supply chain under the DIP policy
$\widehat{\pi }^B$, $\widehat{\pi }^{e}$, $\widehat{\pi }^{r}$, $\widehat{\pi }$	Expected profit of the buyer, expedited and regular suppliers, total supply chain under the TBS policy

We consider a single-product, multi-period ($t= \left\{ 1,2,\ldots ,T \right\}$), stochastic dual sourcing inventory management problem with one buyer and two suppliers. Each period *t*, the buyer faces stochastic, i.i.d, non-negative, stationary demand $D_t$. The buyer replenishes the same product from two different suppliers: the expedited supplier *e* and the regular supplier *r*. Supplier *e* is the fast and expensive supplier, whereas supplier *r* is the slow and low cost supplier. Supplier *e* is a fast supplier with a replenishment lead time of $l_e$. Supplier *r* is located further away from the buyer with a replenishment lead time of $l_r> l_e$. A unit inventory holding cost *h* is incurred for each unit of inventory at the end of a period and a penalty cost of *b* is incurred for each unit of demand that is not satisfied. Unsatisfied demand is assumed to be backordered. We also assume that the salvage value of the product at the end of the horizon is zero. The buyer sells the product at a unit price of *p* and purchases it at wholesale prices of $w_e$ and $w_r$ from the expedited and regular suppliers respectively. Suppliers *e* and *r* produce (or procure) the product at a unit cost price of $c_e$ and $c_r$ respectively. We assume that $p \ge w_e \ge w_r>\max \{c_e,c_r\}$, $h<b$, and that decisions are made under uncertainty, i.e., $Q_{t}^{e}$, $Q_{t}^{r}$, $\widehat{Q}_{t}^{e}$ and $\widehat{Q}^{r}$ are decided upon before demands get revealed (make-to-stock policy). The exact order of events will be discussed in the next subsections. Also, the two suppliers are assumed to decide on their production quantities after $Q_{t}^{e}$, $Q_{t}^{r}$, $\widehat{Q}_{t}^{e}$ and $\widehat{Q}^{r}$ get revealed (make-to-order policy). The objective of each part of the supply chain is to maximize its expected profit.

### Dual index policy

In this subsection, we will present the order of events and the expected profit function for the buyer (Sect. 3.2.1), for the suppliers (Sect. 3.2.2) and for the total supply chain (Sect. 3.2.3) under the DIP policy.

#### The buyer’s problem

The order of events in a given period *t* is as follows:

- First, the state of the system is updated by inspecting the on-hand inventory $I_t$, expedited orders placed in the past $l_e$ periods, and the regular orders placed in the part $l_r$ periods. Then, the expedited and regular inventory positions are calculated as follows:
  $$\begin{aligned} IP_t^e= & {} I_t+(Q_{t-l_e}^e+\cdots +Q_{t-1}^e)+(Q_{t-l_r}^r+\cdots +Q_{t-\Delta l-1}^r), \\ IP_t^r= & {} I_t+(Q_{t-l_e}^e+\cdots +Q_{t-1}^e)+(Q_{t-l_r}^r+\cdots +Q_{t-1}^r). \end{aligned}$$
- The decisions $Q_t^e$ and $Q_t^r$ are made based on the expedited and regular inventory positions, respectively. The expedited order $Q_t^e$ is added to $IP_t^r$ before $Q_t^r$ is determined.
- The regular order $Q_{t-l_r}^r$ and expedited order $Q_{t-l_e}^e$ physically arrive.
- Demand $D_t$ is revealed and satisfied if sufficient on-hand inventory is available. Otherwise, excess demand is backordered.
- The inventory levels are updated and holding or penalty costs are incurred.

In each period *t*, the expedited inventory position may exceed the target basestock level $Y^e$ due to $Q_{t-\Delta l}^r$, resulting in the overshoot $O_t =(IP_t^e+Q_{t-\Delta l}^r-Y^e)^+$. In this case, $Q_t^e=0$. Otherwise, in case $IP_t^e+Q_{t-\Delta l}^r < Y^e$, then a positive expedited order $Q_t^e =(Y^e-IP_t^e-Q_{t-\Delta l}^r)^+$ is made to raise the inventory position to $Y^e$. The system recursions and dynamics are:

$$\begin{aligned} IP_{t+1}^e &= IP_{t}^e +Q_t^e +Q_{t-\Delta l}^r -D_t= Y^e+O_t-D_t,\\ IP_{t+1}^r&= IP_{t}^r +Q_t^e +Q_{t}^r -D_t =Y^r -D_t, \\ I_{t+1}& = I_t + Q_{t-l_e}^e +Q_{t-l_r}^r -D_t, \end{aligned}$$

and the expedited and regular orders are given by:

$$\begin{aligned} Q_t^e&=(Y^e-IP_t^e-Q_{t-\Delta l}^r)^+, \\ Q_t^r&= Y^r -(IP_t^r+Q_{t}^e)=D_{t-1}-Q_t^e. \end{aligned}$$

The holding and penalty costs are charged on the excess on-hand inventory $I_t^+=\max (I_t,0)$ and backorder quantity $I_t^+=\max (-I_t,0)$, respectively. The buyer’s profit in period *t* is computed as:

$$\begin{aligned} \pi _t^B = pD_t - h I_t^+ -bI_t^- -\Delta w Q_t^e -w_r D_{t-1}. \end{aligned}$$

Consequently, the expected profit of the buyer becomes:

1 $$\begin{aligned} \pi ^B = \lim \limits _{T\rightarrow \infty }\frac{1}{T}\sum \limits _{t=1}^T \pi _t^B=(p-w_r)\mu - h E[I^+] -bE[I^-] -\Delta w E[Q^e], \end{aligned}$$

where $I^+$ and $I^-$ are the stationary excess inventory and backorder quantity. As shown by Veeraraghavan and Scheller-Wolf (2008), the overshoot distribution $O_t$ and the expedited order $Q_t^e$ are only functions of $\Delta =Y^r-Y^e$, independent of $Y^e$. Also, $I_{t+1}=Y^e+O_{t-l_e}-D_{t}^{-l_e}$, where $D_{t}^{-l_e}= D_{t}+D_{t-1}+\cdots D_{t-l_e}$. Let $H_{t,\Delta }$ and $F_{t,l_e}$ denote the cumulative distribution function of $O_{t-l_e}$ and $D_{t}^{-l_e}$ with stationary versions $H_{\Delta }$ and $F_{e}$. As shown in “Appendix 1”, the expected profit of the buyer can be then expressed as:

2 $$\begin{aligned} \pi ^B&= (p-w_r)\mu -\Delta w E[Q^e]-h L_1(Y^{e^*})-b L_2(Y^{e^*})\nonumber \\ & \quad -\int _0^{\infty } \left[ (h+b)F_e(Y^{e^*}+x)-b\right] [1-H_{\Delta }(x)]dx, \end{aligned}$$

with $L_1(y) =\int _0^y (y-z)dF_e(z)$, $L_2(y) =\int _y^\infty (z-y)dF_e(z)$ and $Y^{e^*}$ the optimal expedited basestock level that solves $\int _0^\infty F_e(Y^e+x)dH_{\Delta }(x)= \frac{b}{b+h}$ (see “Appendix 1”).

Taking derivative with respect to $\Delta$, we obtain:

$$\begin{aligned} \frac{\partial \pi ^B}{\partial \Delta }=-\Delta w \frac{\partial E[Q^e]}{\partial \Delta }+\int _0^\infty \left[ (h+b)F_e(Y^{e^*}+x)-b\right] \frac{\partial H_{\Delta }(x)}{\partial \Delta } dx. \end{aligned}$$

The optimal $\Delta ^*$ solves

3 $$\begin{aligned} -\Delta w \frac{\partial E[Q^{e^*}]}{\partial \Delta }+\int _0^\infty [(h+b)F_e(Y^{e^*}+x)-b]\frac{\partial H_{\Delta ^*}(x)}{\partial \Delta } dx=0. \end{aligned}$$

Using the profit of each $(\Delta ,Y^{e^*}(\Delta ))$ pair, the maximum profit pair can be found by a one-dimensional search over $\Delta$. This yields the optimal $\Delta ^*$ and the optimal dual-index policy parameters $Y^{e^*}(\Delta ^*)$ and $Y^{r^*}(\Delta ^*)= \Delta ^*+Y^{e^*}(\Delta ^*)$.

#### The suppliers’ profit functions

The expected profit of the expedited supplier *e* is:

4 $$\begin{aligned} \pi ^e =(w_e-c_e)E[Q^{e^*}], \end{aligned}$$

and the expected profit of the regular supplier *r* is:

5 $$\begin{aligned} \pi ^r =(w_r-c_r)(\mu -E[Q^{e^*}]). \end{aligned}$$

#### The total supply chain profit

The total expected profit of the supply chain is given by:

6 $$\begin{aligned} \pi& =\pi ^B + \pi _e+ \pi _r =(p-w_r)\mu -\Delta w E[Q^e]-h L_1(Y^{e^*})-b L_2(Y^{e^*})\nonumber \\ & \quad -\int _0^{\infty } \left[ (h+b)F_e(Y^{e^*}+x)-b\right] [1-H_{\Delta }(x)]dx \nonumber \\{} & \quad +(w_e-c_e)E[Q^{e}]+(w_r-c_r)(\mu -E[Q^{e}]) \nonumber \\&=(p-c_r)\mu -\Delta c E[Q^e]-h L_1(Y^{e^*})-b L_2(Y^{e^*})\\ & \quad -\int _0^{\infty } \left[ (h+b)F_e(Y^{e^*}+x)-b\right] [1-H_{\Delta }(x)]dx. \end{aligned}$$

### Tailored base-surge policy

In this subsection, we present the order of events and the expected profit under the TBS policy for the buyer in (Sect. 3.3.1) for the suppliers in (Sect. 3.3.2) and for the total supply chain (Sect. 3.3.3).

#### The buyer’s problem

The order of events in a given period *t* is as follows:

- First, the state of the system is updated by inspecting the on-hand inventory $I_t$, expedited orders placed in the past $l_e$ periods, and the regular orders placed in the past $l_r$ periods. Then, the expedited inventory position is calculated as follows:
  $$\begin{aligned} \widehat{IP}_t^e& = I_t+(\widehat{Q}_{t-l_e}^e+\cdots +\widehat{Q}_{t-1}^e)+(t-\Delta l-1 -(t-l_r)+1)\widehat{Q}^r \\&= I_t+(\widehat{Q}_{t-l_e}^e+\cdots +\widehat{Q}_{t-1}^e)+(l_e)\widehat{Q}^r. \end{aligned}$$
- Decisions $\widehat{Q}_t^e$ and $\widehat{Q}^r$ are made.
- The regular order $\widehat{Q}^r$ and expedited order $\widehat{Q}_{t-l_e}^e$ physically arrive.
- $D_t$ is revealed and satisfied if sufficient on-hand inventory is available. Otherwise, excess demand is backordered.
- The inventory levels are updated and holding or penalty costs are incurred.

In each period *t*, the expedited inventory position may exceed the target expedited basestock level $\widehat{Y}$ due to the standing order $\widehat{Q}^r$, resulting in the overshoot $\widehat{O}_t =(\widehat{IP}_t^e+\widehat{Q}^r-\widehat{Y})^+$. In this case, $\widehat{Q}_t^e=0$. Otherwise, in case $\widehat{IP}_t^e+\widehat{Q}^r < \widehat{Y}$, then a positive expedited order $\widehat{Q}_t^e =(\widehat{Y}-\widehat{IP}_t^e-\widehat{Q}^r)^+$ is made to raise the inventory position to $\widehat{Y}$.

The buyer’s profit in period *t* is:

$$\begin{aligned} \widehat{\pi }_t^B = p D_t - h I_t^+ -bI_t^- -w_e \widehat{Q}_t^e -w_r \widehat{Q}^r. \end{aligned}$$

Like under the DIP policy and following Janakiraman et al. (2014), the overshoot distribution $\widehat{O}_t$ and the expedited order $\widehat{Q}_t^e$ are only functions of $\widehat{Q}^r$, independent of $\widehat{Y}$. In addition, $I_{t+1}=\widehat{Y}+\widehat{O}_{t-l_e}-D_{t}^{-l_e}$. Let $\widehat{H}_{t,\widehat{Q}^r}$ denote the cumulative distribution function of $\widehat{O}_{t-l_e}(\widehat{Q}^r)$ with stationary version $\widehat{H}_{\widehat{Q}^r}$. Similar to the DIP case, the expected profit of the buyer can be expressed as:

7 $$\begin{aligned} \widehat{\pi }^B= & {} (p-w_e)\mu +\Delta w \widehat{Q}^r -h L_1(\widehat{Y}^*)-b L_2(\widehat{Y}^*)\nonumber \\{} & {} -\int _0^{\infty } \left[ (h+b)F_e(\widehat{Y}^*+x)-b\right] \left[ 1-\widehat{H}_{\widehat{Q}^r}(x)\right] dx, \end{aligned}$$

with $\widehat{Y}^*$ the optimal expedited basestock level that solves $\int _0^{\infty }F_e(\widehat{Y}+x)d\widehat{H}_{\widehat{Q}^r}(x) = \frac{b}{b+h}$. One deduces from the result of Janakiraman et al. (2014), that function $\widehat{\pi }^B$ is concave in $\widehat{Q}^r$. Taking derivative with respect to $\widehat{Q}^r$, we obtain:

$$\begin{aligned} \frac{\partial \widehat{\pi }^B}{\partial \widehat{Q}^r}=\Delta w +\int _0^\infty \left[ (h+b)F_e(\widehat{Y}^*+x)-b\right] \frac{\partial \widehat{H}_{\widehat{Q}^r}(x)}{\partial \widehat{Q}^r} dx. \end{aligned}$$

The optimal regular order $\widehat{Q}^{r^*}$ solves

8 $$\begin{aligned} \Delta w +\int _0^\infty \left[ (h+b)F_e(\widehat{Y}^*+x)-b\right] \frac{\partial \widehat{H}_{\widehat{Q}^{r^*}(x)}}{\partial \widehat{Q}^r} dx=0. \end{aligned}$$

Using the profit of each $(\widehat{Q}^r,\widehat{Y}^{*}(\widehat{Q}^r))$ pair, we find the maximum profit pair by a one-dimensional search over $\widehat{Q}^r$. This yield the optimal standing order $\widehat{Q}^{r^*}$ and the optimal $\widehat{Y}^{*}(\widehat{Q}^{r^*})$.

#### The suppliers’ profits

The expected profit of the expedited supplier *e* is:

9 $$\begin{aligned} \widehat{\pi }^e =(w_e-c_e)(\mu -\widehat{Q}^{r^*}), \end{aligned}$$

and the expected profit of the regular supplier *r* is given by:

10 $$\begin{aligned} \widehat{\pi }^r =(w_r-c_r)\widehat{Q}^{r^*}. \end{aligned}$$

#### The total supply chain profit

The total expected profit of the supply chain is given by:

11 $$\begin{aligned} \widehat{\pi }&= \widehat{\pi }^B + \widehat{\pi }_e+ \widehat{\pi }_r = (p-w_e)\mu +\Delta w \widehat{Q}^r -h L_1(\widehat{Y}^*)-b L_2(\widehat{Y}^*)\nonumber \\{} & {} \quad -\int _0^{\infty } \left[ (h+b)F_e(\widehat{Y}^*+x)-b\right] \left[ 1-\widehat{H}_{\widehat{Q}^r}(x)\right] dx \nonumber \\{} & {}\quad +(w_e-c_e)(\mu -\widehat{Q}^{r^*})+(w_r-c_r)\widehat{Q}^{r^*} \nonumber \\&= (p-c_e)\mu +\Delta c \widehat{Q}^r -h L_1(\widehat{Y}^*)-b L_2(\widehat{Y}^*)\nonumber \\{} & {}\quad -\int _0^{\infty } \left[ (h+b)F_e(\widehat{Y}^*+x)-b\right] \left[ 1-\widehat{H}_{\widehat{Q}^r}(x)\right] dx. \end{aligned}$$

##### Remark 1

If we would like to explicitly consider the suppliers’ decision making, then we need to assume that there is a central decision authority. In that case, the achieved profit would form an upper bound for the supply chain profit to be achieved under decentralized decision making.

For DIP, using Equation (3), the optimal $\Delta ^*$ for the centralized system would solve

$$\begin{aligned} -\Delta c \frac{\partial E[Q^{e^*}]}{\partial \Delta }+\int _0^\infty \left[ (h+b)F_e(Y^{e^*}+x)-b\right] \frac{\partial H_{\Delta ^*}(x)}{\partial \Delta } dx=0. \end{aligned}$$

For TBS, using Equation (8), the optimal $\widehat{Q}^{r^*}$ would solve

$$\begin{aligned} \Delta c +\int _0^\infty [(h+b)F_e(\widehat{Y}^*+x)-b]\frac{\partial \widehat{H}_{\widehat{Q}^{r^*}(x)}}{\partial \widehat{Q}^r} dx=0. \end{aligned}$$

Note that this is equivalent to the buyer problem with $\Delta c$ replacing $\Delta w$. So solving the centralized approach would have been easy but unrealistic. Hence, a complex problem under the decentralized system should be investigated to understand the interaction and competitions between regular and emergency suppliers in addition to identifying the incentives needed for all parties to achieve an enhanced performance. This problem is outside the scope of the current paper but is an interesting problem to consider in future research.

## Analytical and numerical results

In this section, we first present our analytical results of the effects of several model parameters on the expected profits of the buyer, suppliers and total supply chain. The proofs are in “Appendix 2”.

### Lemma 1

**Effects of model parameters**

(i) $\widehat{\pi }^r$ is increasing in $w_e$; $\pi ^B$ and $\widehat{\pi }^B$ are decreasing in $w_e$.
(ii) $\pi ^B$ and $\widehat{\pi }^B$ are decreasing in $w_r$; $\widehat{\pi }^e$ is increasing in $w_r$.
(iii) $\pi ^e$, $\widehat{\pi }^e$, $\pi$ and $\widehat{\pi }$ are decreasing in $c_e$.
(iv) $\pi ^r$, $\widehat{\pi }^r$, $\pi$ and $\widehat{\pi }$ are decreasing in $c_r$.
(v) $\pi ^B$ and $\widehat{\pi }^B$ are decreasing in *h*.
(vi) $\pi ^B$ and $\widehat{\pi }^B$ are decreasing in *b*.

However, the most crucial parameter for our study is the lead time difference. Deriving exact results in terms of the lead time difference is mathematically complex. Therefore, we conduct numerical studies to determine the impact of the lead time difference on the system performance in terms of profit. In addition, we conduct a sensitive analysis in this matter. In all our numerical studies, the performance of both policies is measured by the expected total supply chain profit. The parameters that have been varied are the following:

- $$\Delta l$$ = [1,10]
- $$CV(D_t)$$ = {0.25,0.5,1,2}
- $$w_r,w_e,c_r,c_e$$ = {1,2,4,6,8,10}
- *b* = {2,5,10,20,50,100}
- *h* = {1,2,4,6,8,10}

In all experiments, we assume that $w_e \ge w_r \ge c_r$, $w_e \ge c_e$, and $b>h$. $D_t$ has been assumed to follow the Gamma distribution. The following parameters have been fixed in all experiments: $\mu =10$, $p=15$, and $l_e=0$. Hence, $\Delta l = l_r$. The policy parameters have been optimised via a simulation-based optimisation procedure for which the interior point algorithm in MATLAB has been used. We investigated a large test bed of 225 different combinations of the parameters shown above. See Table 2 in “Appendix 3” for an overview of the results of all experiments and the input parameters.

Looking at the results, we find in general that when $\Delta l$ is small, DIP typically outperforms, whereas when $\Delta l$ grows large, TBS mostly outperforms. This is for a single-echelon system in line with what we know from the literature. In our experiments, we are mainly interested in the unique value of $\Delta l$ where the policy dominance swaps, which we call the *turning point*. Therefore, we define the following two turning points:

- $\delta l_{sc}$: the smallest (integer) lead time difference ($\Delta l$) where TBS becomes the preferred policy from a supply chain perspective;
- $\delta l_b$: the smallest (integer) lead time difference ($\Delta l$) where TBS becomes the preferred policy from the buyer’s perspective.

In Sect. 4.1, we present a base case, which is an illustrative example. Then, the summary of different experiments are presented in Sect. 4.2. In Sect. 4.3, we discuss the case where the entire supply chain is centrally controlled by one decision authority.

**Fig. 1:**
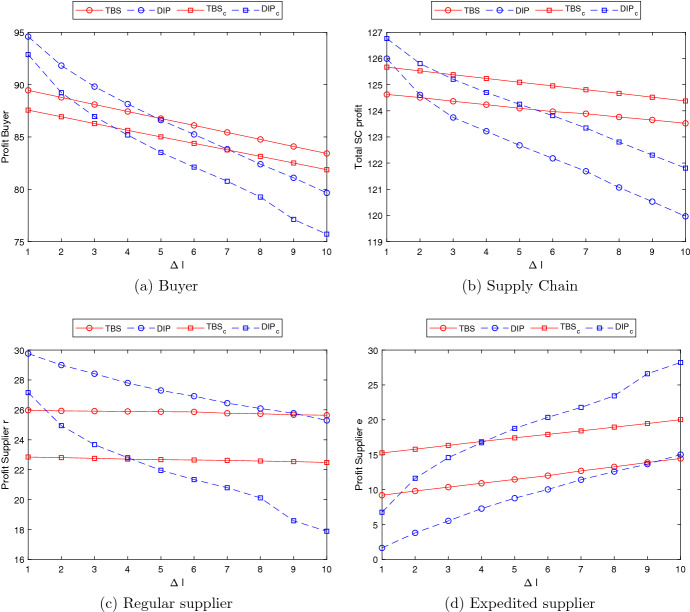
Profit functions for the buyer, supply chain, and the two suppliers as function of $\Delta l$

### Base case

The base case is the experiment with the following parameter values: $h=1$, $b=10$, $w_e=8$, $w_r=4$, $c_e=2$, $c_r=1$, and $CV(D_t)=0.5$. Figure 1 shows the results for the base case where the functions *TBS* and *DIP* show the results for the problem as described in Sect. 3. The functions $TBS_c$ and $DIP_c$ show the results for the case the entire supply chain is centrally controlled by one decision authority, which will be explained in more detail in Sect. 4.3. Looking at *TBS* and *DIP* functions in Fig. 1, it becomes clear that for the buyer, $\delta l_b = 5$ (Fig. 1a), whereas $\delta l_{sc} =3$ (Fig. 1b). This result of $\delta l_{sc} < \delta l_b$ is exemplary for the results of our experimental study. It is an interesting result, because it shows that when the supply chain perspective is taken into consideration, the TBS policy becomes dominant after a relatively shorter lead time difference. For the sake of completeness, we also present the profit functions of the regular supplier (Fig. 1c) and the expedited supplier (Fig. 1d). For both supplier, it holds that their turning points are achieved at relatively large $\Delta l$ with the difference that the regular supplier prefers TBS only when $\Delta l$ grows large, whereas the opposite holds for the expedited supplier.

### Varying several parameters

**Fig. 2:**
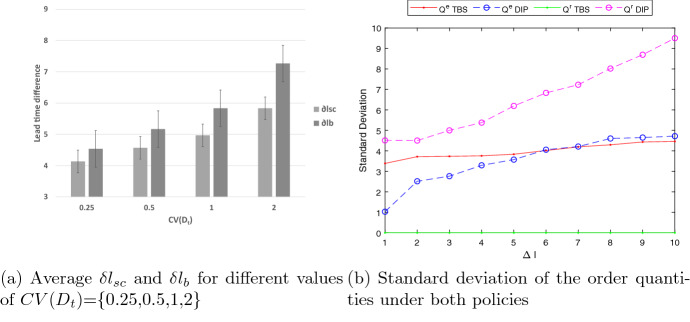
**a** Average $\delta l_{sc}$ and $\delta l_{b}$ for different values of $CV(D_t)=\{0.25,0.5,1,2\}$.** b** Standard deviation of the order quantities under both policies

In the base case, we assumed $CV(D_t) = 0.5$. Figure 2a shows the average $\delta l_{sc}$ and $\delta l_{b}$ (and their standard errors) for all experiments that we conducted for different values of $CV(D_t)$. The result shows that both $\delta l_{sc}$ and $\delta l_{b}$ increase when the level of demand uncertainty increases. In other words, the higher the level of demand uncertainty, the larger the turning points ($\delta l_{sc}$ and $\delta l_{b}$). However, $\delta l_{sc}$ remains moderately smaller than $\delta l_{b}$ with an increasing difference in $CV(D_t)$. The results show that when the supply chain perspective is taken, TBS outperforms after a shorter lead time difference. That means that the flexibility offered by DIP is only of added value when the lead time difference between the two suppliers is short. Once the lead time difference exceeds the unique turning point, the order stability of TBS becomes of higher added value.

**Fig. 3:**
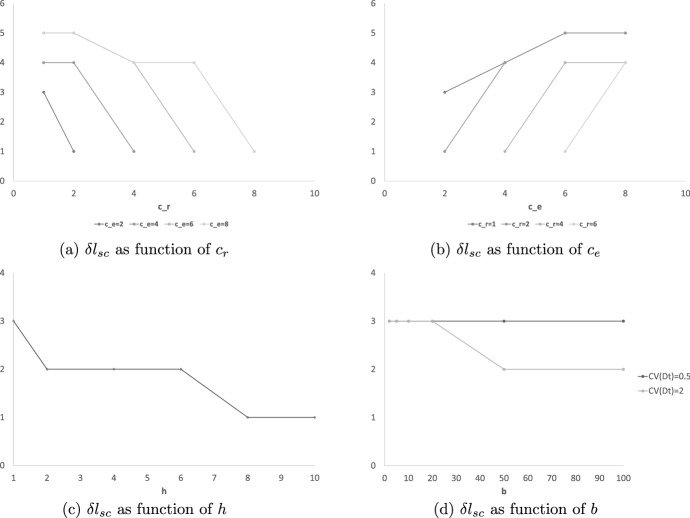
$\delta l_{sc}$ as function of several model parameters ($w_e=10$, $w_r=8$)

Figure 3 shows the results when the unit costs $c_e$ and $c_r$ are varied (in case $w_e=10$, $w_r=8$). The results of other experiments (for other values of $w_e$ and $w_r$), which we do not display here due to space limitations, are very similar. The results show that $\delta l_{sc}$ is decreasing in the unit cost of the regular supplier (Fig. 3a). In other words, the larger the unit cost of the regular supplier (while fixing all other parameters), the faster TBS becomes the preferred policy. The opposite holds for the unit cost of the expedited supplier, which results in an increased turning point (Fig. 3b).

Increasing the unit holding cost *h* results in a decrease of $\delta l_{sc}$ (Fig. 3c). However, $\delta l_{sc}$ seems to be insensitive to the unit backorder cost *b* (Fig. 3d). Only at high levels of $CV(D_t)$, $\delta l_{sc}$ slightly decreases at high values of *b*. The two policies do not only impact the expected order quantities towards the two suppliers, but also the variance of the orders. Figure 2b shows the standard deviation of the order quantities towards both suppliers under the two policies as function of $\Delta l$. One can observe that as $\Delta l$ increases, the emergency supplier faces a lower order variability under TBS as compared to DIP.

### Central decision authority

In Sect. 3, we presented the model formulation for the case where the buyer makes the order decisions with the main objective to study how those decisions affect the suppliers’ and total supply chain profits. The results of the numerical experiments for that case have been presented in Sects. 4.1 and 4.2. In addition, we run experiments where we assumed that there is one central decision authority for the entire supply chain that makes order decisions such that the total supply chain profit is maximized rather than the buyer’s profit. Figure 1 shows also the results of these experiments for the base case, which are denoted by the $TBS_c$ and $DIP_c$ functions. The results show that the total supply chain profit increases under centralized control (Fig. 1b), which is due to the fact that the double marginalization effect has been eliminated (Li et al. 2013). We also compare the profits of each supply chain party under centralized control given the same $w_e$ and $w_r$ values that have been used for the base case experiment. The results show that the buyer (Fig. 1a) and regular supplier’s profits (Fig. 1c) are lower under centralized control, whereas the expedited supplier’s profit (Fig. 1d) is higher under centralized control. The reason is that under centralized control, the cost difference $\Delta c = c_e - c_r$ is smaller compared to the wholesale price difference $\Delta w = w_e - w_r$ under decentralized control, which results in a larger order for the expedited supplier (and lower quantity for the regular supplier). In other words, the smaller the sourcing cost difference, the more is ordered from the more expensive, but responsive supplier.

## Data collection and managerial insights

In order to have a better understanding of the real-life implications of our study, we conducted an empirical study by collecting relevant data from 51 manufacturing companies that have a manufacturing facility in the United Arab Emirates (UAE). Almost all these companies apply a dual- or multi-sourcing strategy for their critical products. The data collection occurred by interviewing the logistics, supply chain or procurement manager within the company. The data collection allowed us to get several insights from real-life practices, in particular related to the responsiveness of suppliers (in terms of the lead times) and the relative cost difference between different suppliers. Table 1 shows in which industries the interviewed companies are active. Unfortunately, we are not able to disclose the names of the companies, as we agreed with them, due to confidentiality reasons.

**Table 1:** Industries in which the interviewed companies are active

Industry	Frequency
Primary metal & construction	11
Oil & Gas	8
Chemicals	7
Food & household products	6
Automotive	4
Retail	4
Energy equipment	3
Packaging	3
Textile & luxury goods	3
IT	2
	51

**Fig. 4:**
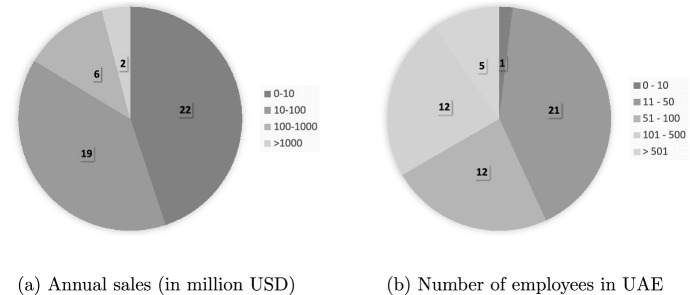
Annual sales (in million USD) and the number of employees (in UAE) of the interviewed companies

To get an impression of the size of the interviewed companies, Fig. 4a shows the annual sales and Fig. 4b shows the number of employees that are based in the UAE. Kindly note that two firms did not disclose their sales.

**Fig. 5:**
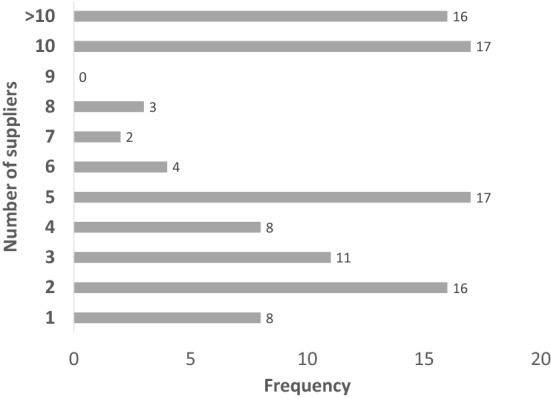
Total number of suppliers for the two critical products

We asked the interviewee to identify the two most critical items that are input to their production process. These items can be raw materials or semi-finished products that are purchased from (an) external supplier(s). Figure 5 shows the number of different suppliers from where those two products are (or could be) ordered. The suppliers are located in: UAE, EU (mainly UK, Switzerland, Germany, and the Netherlands), GCC (Saudi-Arabia and Oman), China, Japan, USA, Canada, Brazil, South Africa, Thailand, India, Turkey, Singapore, Indonesia, Taiwan, India, and South Korea. Hence, the suppliers are well spread over the world.

**Fig. 6:**
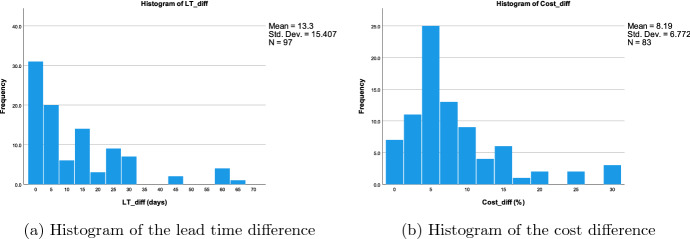
Histograms of the lead time (in days) and cost differences (in %) between suppliers

We also asked the respondents about the lead times of the two critical items and the relative cost difference between the suppliers. Figure 6a shows the distribution of the lead time differences (with an average of about 2 weeks) and Fig. 6b shows the distribution of the relative purchase cost difference between the suppliers (with an average of about 8% difference). From Fig. 6a, it seems that a substantial part of the lead time differences will likely be large enough such that TBS may be justified as a serious alternative.

### Managerial insights

Our analytical results show that the regular supplier’s profit is increasing in the emergency supplier’s wholesale price and decreasing in its own unit purchasing cost. In other words, the more the emergency supplier charges the buyer and the less the regular supplier’s purchasing cost is, the more beneficial for the regular supplier. On the other hand, the emergency supplier’s profit is decreasing in both the emergency supplier’s wholesale price and in its own unit purchasing cost.

From the literature, we learn that for a single-echelon system, i.e., for the buyer only, TBS policy is asymptotically optimal (see Sect. 2). That means that TBS is optimal when the lead time difference between the two suppliers grows to infinity. When looking at a more extended system, like the two-echelon system that we consider in our study, we find from our numerical results that TBS outperforms DIP after a very limited lead time difference, typically when the lead time difference is around 4 periods (on average 3.84 periods over all our numerical experiments). The lead time data that we collected from the interviewed firms shows that 66% of the lead time differences are at least 4 periods (see Fig. 6a). Our numerical experiments show that the turning point, which is the lead time difference from where TBS outperforms, is dependent on several model parameters. The turning point increases in the level of demand uncertainty and the expedited supplier’s unit (purchasing) cost and it decreases in the regular supplier’s (purchasing) cost and the unit inventory holding cost. In the current situation, none of the interviewed managers indicated that TBS policy was implemented, and therefore, the TBS policy may become a beneficial policy for a substantial part of the interviewed manufacturing firms. It is likely that the collected data from the interviewed companies are representative for many manufacturing firms, which may indicate that the results of our study may be of interest for a larger group of manufacturing industries. Hence, based on our experimental results, we would recommend firms having a lead time difference of more than 4 periods to consider implementing the TBS policy. Otherwise, if the lead time difference is shorter (less than 4 time periods), then we would recommended to consider implementing the DIP policy.

## Conclusions and future research

In this paper, we study a stochastic dual sourcing system. Under dual sourcing, a buyer can get inventory replenished from a fast and expensive (expedited) supplier and from a slow and lower cost (regular) supplier. Equivalently, the inventory can be replenished from one supplier by using two different transportation modes. Dual sourcing systems have been widely studied in the literature, but mostly in a single-echelon setting and under the assumption of consecutive lead time difference. We study the dual sourcing problem from a supply chain perspective, i.e., by explicitly taking into consideration the effect of the control policies on the entire supply chain, including the suppliers. Also, we consider the general (nonconsecutive) lead time case. The latter significantly complicates the dual sourcing problem. From previous studies, we learn that for a single-echelon system, DIP is optimal if the lead time difference is one period and TBS becomes optimal when the lead time difference goes to infinity. In this study, we compare the performance of both policies and one of the main findings is that TBS outperforms DIP after a lead time difference of a few time periods, which is for many supply chains a very realistic setting, as our collected data confirm. The flexibility that DIP offers in terms of adjusting order quantities appears to be only valuable when lead time difference is short, whereas the added value of the stability offered by TBS prevails when the lead time difference exceeds a certain turning point. The exact turning point, which is the lead time difference where TBS starts to outperform, is dependent on the suppliers’ and the buyer’s cost structure and on the level of demand uncertainty, as our numerical experiments show. However, in all scenarios that we evaluated numerically, the turning point is always limited to a few time periods. This result is not only interesting from a theoretical perspective, but it can also have serious implications for many supply chains, given the simple and appealing structure of the TBS policy for the regular suppliers. Under TBS, the regular supplier gets every period a fixed order quantity, which allows that supplier to operate highly efficiently and in turn increase the service level at lower cost.

The results from this study inspires several ideas to address in future research. First, our study was limited to a two-echelon system. Since moving from a single-echelon to a two-echelon system resulted in a significant decrease of the turning point, it could be that considering a system with more echelons will further lower the turning point. If that happens, it means that TBS is not asymptotically optimal in the lead time, but also in the network size of the supply chain. This is worth investigating given that supply chains in real-life are very fragmented and consist of many echelons. Second, in our modeling approach, we assumed a stationary demand distribution. Many supply chains are often exposed to disruptions, as the current COVID-19 clearly showed, which makes it interesting to study how these dual sourcing policies would perform under disrupted supply and demand. Third, if TBS is implemented, the buyer may require a compensation (lower unit price) for ordering a fixed quantity from the regular supplier. Hence, interesting price negotiations may follow and it would be worth to investigate whether supply chain coordination and a price equilibrium can be achieved and under which conditions. Fourth, comparing our results with other dual sourcing approaches in the literature may result in more valuable managerial insights.

## Data Availability

The datasets generated during the current study are available from the corresponding author on reasonable request.

## Appendix 1: Buyer’s expected profit $\pi ^B$

Under the DIP policy, the expected profit of the buyer is:

$$\begin{aligned} \pi ^B=(p-w_r)\mu -\Delta w E[Q^e]-h E\{I^+\}-b E\{I^-\}. \end{aligned}$$

Following Veeraraghavan and Scheller-Wolf (2008), the expected profit can be seen as:

$$\begin{aligned} \pi ^B=(p-w_r)\mu -\Delta w E[Q^e]-h E\{L_1(Y^e+O(\Delta ))\}-b E\{L_2(Y^e+O(\Delta ))\}, \end{aligned}$$

where *Y* is the the expedited basestock level, $L_1(y)=E\{(y-\sum _{i=1}^{\ell _e+1}D_i)^+\}$, $L_2(y)=E\{(\sum _{i=1}^{\ell _e+1}D_i-y)^+\}$, $l_e$ is the lead time from the expedited supplier to the buyer, and $O(\Delta )$ is the stationary overshoot associated to $\Delta$. Since $F_e$ is the distribution of $\sum _{i=1}^{\ell _e+1}D_i$, we have, $L_1(y)=\int _0^y(y-t)dF_e(t)$ and $L_2(y)=\int _y^\infty (t-y)dF_e(t)$. Easy computations show that $L_1'(y)=F_e(y)\ge 0$, $L_2'(y)=F_e(y)-1\le 0$ and $L_2(y)=L_1(y)-y+(\ell _e+1)\mu .$

Knowing that $H_{\Delta }$ is the stationary distribution function of the overshoot $O(\Delta )$, the expected profit can be re-written as:

$$\begin{aligned} \pi ^B&= (p-w_r)\mu -\Delta w E[Q^e]-h \int _0^\infty L_1(Y^e+x)dH_{\Delta }(x)\\{} & \quad -b \int _0^\infty L_2(Y^e+x)dH_{\Delta }(x). \end{aligned}$$

Taking derivative with respect to $Y^e$, the optimal basetock level $Y^{e^*}$ solves

$$\begin{aligned} \frac{\partial \pi ^B}{\partial Y^e}=-(h+b)E\{F_e(Y^e+O(\Delta ))\}+b=0. \end{aligned}$$

If we denote $M_{\Delta }(Y^e)=E\{F_e(Y^e+O(\Delta ))\}=\int _0^\infty F_e(Y^e+x)dH_{\Delta }(x)$, then the optimal basestock level $Y^{e^*}$ solves $M_{\Delta }(Y^{e*})=b/(b+h)$. Replacing $Y^{e*}$ in the profit expression yields

12 $$\begin{aligned} \pi ^B=(p-w_r)\mu -\Delta w E[Q^e]-h \int _0^\infty L_1(Y^{e^*}+x)dH_{\Delta }(x)-b \int _0^\infty L_2(Y^{e^*}+x)dH_{\Delta }(x). \end{aligned}$$

An integration by part shows that

13 $$\begin{aligned} \pi ^B& = (p-w_r)\mu -\Delta w E[Q^e]-h L_1(Y^{e^*})-b L_2(Y^{e^*})\nonumber \\{} & \quad -\int _0^\infty \left[ (h+b)F_e(Y^{e^*}+x)-b\right] [1-H_{\Delta }(x)]dx. \end{aligned}$$

## Appendix 2: Proof of Lemma 1

(i) From Equation (8), we have:
  $$\begin{aligned} \frac{\partial \widehat{\pi }^B}{\partial \widehat{Q}^{r}} = w_e-w_r + \int _{0}^{\infty }\left[ (h+b)F_e (\widehat{Y}^*+x)-b\right] \frac{\partial \widehat{H}_{\widehat{Q}^{r}}(x)}{\partial \widehat{Q}^{r}} dx = 0 \end{aligned}$$
  That implies:
  $$\begin{aligned} \frac{\partial ^2 \widehat{\pi }^B}{\partial w_e\partial \widehat{Q}^{r}}= {} 1 +\frac{\partial ^2 \widehat{\pi }^B}{\partial ^2 \widehat{Q}^{r}}\frac{\partial \widehat{Q}^{r^*}}{\partial w_e}=0. \Rightarrow \frac{\partial \widehat{Q}^{r^*}}{\partial w_e}= {} \frac{-1}{\frac{\partial ^2 \widehat{\pi }^B}{\partial ^2 \widehat{Q}^{r}}}>0 \text{(since } \frac{\partial ^2 \widehat{\pi }^B}{\partial ^2 \widehat{Q}^{r}}<0 \text{ because } \widehat{\pi }^B \text{ is } \text{ concave } \text{ in } \widehat{Q}^{r}\text{) }. \end{aligned}$$
  Taking derivatives of $\pi ^B$, $\widehat{\pi }^B$ and $\widehat{\pi }^r$ with respect to $w_e$, we get:
  $$\begin{aligned}{} & {} \frac{\partial \pi ^B}{\partial w_e} =-E[Q^{e^*}]<0.\\{} & {} \frac{\partial \widehat{\pi }^B}{\partial w_e} =\widehat{Q}^{r^*}-\mu <0.\\{} & {} \frac{\partial \widehat{\pi }^r}{\partial w_e} = (w_r-c_r)\frac{\partial \widehat{Q}^{r^*}}{\partial w_e} >0. \end{aligned}$$
(ii) From Equation (8), we have:
  $$\begin{aligned} \frac{\partial ^2 \widehat{\pi }^B}{\partial w_r\partial \widehat{Q}^{r}} =-1+\frac{\partial ^2 \widehat{\pi }^B}{\partial ^2 \widehat{Q}^{r}}\frac{\partial \widehat{Q}^{r^*}}{\partial w_r}=0 \Rightarrow \frac{\partial \widehat{Q}^{r^*}}{\partial w_r} < 0. \end{aligned}$$
  Taking derivatives of $\pi ^B$, $\widehat{\pi }^B$ and $\widehat{\pi }^e$ with respect to $w_r$, we get:
  $$\begin{aligned}{} & {} \frac{\partial \pi ^B}{\partial w_r} =E[Q^{e^*}]-\mu<0.\\{} & {} \frac{\partial \widehat{\pi }^B}{\partial w_r} =-\widehat{Q}^{r^*} <0.\\{} & {} \frac{\partial \widehat{\pi }^e}{\partial w_r} = -(w_e-c_e)\frac{\partial \widehat{Q}^{r^*}}{\partial w_r} >0. \end{aligned}$$
(iii) From Equation (3) we have:
  $$\begin{aligned} \frac{\partial \pi ^B}{\partial \Delta }=-\Delta w \frac{\partial E[Q^e]}{\partial \Delta }+\int _0^\infty \left[ (h+b)F_e(Y^{e^*}+x)-b\right] \frac{\partial H_{\Delta }(x)}{\partial \Delta } dx=0. \end{aligned}$$
  Since Equation (3) is independent of $c_e$ and $c_r$, we have
  $$\begin{aligned} \frac{\partial E[Q^{e^*}]}{\partial c_e} = \frac{\partial E[Q^{e^*}]}{\partial c_r} =0. \end{aligned}$$
  Similarly, from Equation (8), we get:
  $$\begin{aligned} \frac{\partial \widehat{Q}^{r^*}}{\partial c_e} = \frac{\partial \widehat{Q}^{r^*}}{\partial c_r} =0. \end{aligned}$$
  Since $\frac{\partial E[Q^{e^*}]}{\partial c_e} = 0$ and $\frac{\partial \widehat{Q}^{r^*}}{\partial c_e}=0$, $\frac{\partial \pi ^B}{\partial c_e}=\frac{\partial \pi ^r}{\partial c_e}=0$ and $\frac{\partial \widehat{\pi }^B}{\partial c_e}=\frac{\partial \widehat{\pi }^r}{\partial c_e}=0$. Consequently, we obtain:
  $$\begin{aligned} \frac{\partial \pi }{\partial c_e}= & {} \frac{\partial \pi ^e}{\partial c_e}=-E[Q^{e^*}]<0.\\ \frac{\partial \widehat{\pi }}{\partial c_e}= & {} \frac{\partial \widehat{\pi }^e}{\partial c_e}=-\mu +\widehat{Q}^{r^*} <0. \end{aligned}$$
(iv) Similar to $c_e$, we have $\frac{\partial E[Q^{e^*}]}{\partial c_r} = 0$ and $\frac{\partial \widehat{Q}^{r^*}}{\partial c_r}=0$ implying that $\frac{\partial \pi ^B}{\partial c_r}=\frac{\partial \pi ^e}{\partial c_r}=0$ and $\frac{\partial \widehat{\pi }^B}{\partial c_r}=\frac{\partial \widehat{\pi }^e}{\partial c_r}=0$. Therefore:
  $$\begin{aligned} \frac{\partial \pi }{\partial c_r}=\frac{\partial \pi ^r}{\partial c_r}&=E[Q^{e^*}]-\mu<0.\\ \frac{\partial \widehat{\pi }}{\partial c_r}&= \frac{\partial \widehat{\pi }^r}{\partial c_r}=-\widehat{Q}^{r^*} <0. \end{aligned}$$
(v) From Equation (12), taking derivative of the expected buyer profit $\pi ^B$ with respect to *h* leads to:
  $$\begin{aligned} \frac{\partial \pi ^B}{\partial h} = -\int _0^\infty L_1(Y^{e^*}+x) dH_{\Delta }(x) <0. \end{aligned}$$
  Similarly, taking derivative of $\widehat{\pi }^B$ with respect to *h* leads to:
  $$\begin{aligned} \frac{\partial \widehat{\pi }^B}{\partial h} =-\int _0^\infty L_1(Y^*+x) dH_{\widehat{Q}^{r^*}}(x) <0. \end{aligned}$$
(vi) Similar to *h*, taking derivatives of $\pi ^B$ and $\widehat{\pi }^B$ with respect to *b* leads to:
  $$\begin{aligned} \frac{\partial \pi ^B}{\partial b}= & {} -\int _0^\infty L_2(Y^{e^*}+x) dH_{\Delta }(x)<0.\\ \frac{\partial \widehat{\pi }^B}{\partial b}= & {} -\int _0^\infty L_2(Y^*+x) dH_{\widehat{Q}^{r^*}}(x) <0. \end{aligned}$$

## Appendix 3: Results of the numerical experiments

See Table 2.

**Table 2:** Results of all 225 experiments

*h*	*b*	$w_e$	$w_r$	$c_e$	$c_r$	$CV(D_t)$	$\delta l_{sc}$	$\delta l_{b}$	*h*	*b*	$w_e$	$w_r$	$c_e$	$c_r$	$CV(D_t)$	$\delta l_{sc}$	$\delta l_{b}$
1	10	4	2	2	1	0.25	3	4	1	10	8	4	6	6	0.5	1	5
1	10	6	2	2	1	0.25	3	5	1	10	8	6	6	2	0.5	5	4
1	10	6	4	2	1	0.25	3	3	1	10	8	6	6	4	0.5	4	4
1	10	8	2	2	1	0.25	3	6	1	10	8	6	6	6	0.5	1	4
1	10	8	4	2	1	0.25	3	5	1	10	10	2	6	2	0.5	5	7
1	10	8	6	2	1	0.25	3	4	1	10	10	2	6	4	0.5	4	7
1	10	10	2	2	1	0.25	3	6	1	10	10	2	6	6	0.5	2	7
1	10	10	4	2	1	0.25	3	5	1	10	10	4	6	2	0.5	5	6
1	10	10	6	2	1	0.25	3	5	1	10	10	4	6	4	0.5	3	6
1	10	10	8	2	1	0.25	3	3	1	10	10	4	6	6	0.5	2	6
1	10	6	2	4	1	0.25	4	5	1	10	10	6	6	2	0.5	5	5
1	10	6	4	4	1	0.25	4	4	1	10	10	6	6	4	0.5	4	5
1	10	8	2	4	1	0.25	4	5	1	10	10	6	6	6	0.5	1	5
1	10	8	4	4	1	0.25	4	4	1	10	10	8	6	2	0.5	5	4
1	10	8	6	4	1	0.25	4	4	1	10	10	8	6	4	0.5	4	4
1	10	10	2	4	1	0.25	4	6	1	10	10	8	6	6	0.5	1	4
1	10	10	4	4	1	0.25	4	5	1	10	10	2	8	2	0.5	6	7
1	10	10	6	4	1	0.25	4	5	1	10	10	2	8	4	0.5	5	7
1	10	10	8	4	1	0.25	4	3	1	10	10	2	8	6	0.5	4	7
1	10	8	2	6	1	0.25	5	5	1	10	10	2	8	8	0.5	2	7
1	10	8	4	6	1	0.25	5	4	1	10	10	4	8	2	0.5	6	6
1	10	8	6	6	1	0.25	5	3	1	10	10	4	8	4	0.5	5	6
1	10	10	2	6	1	0.25	5	6	1	10	10	4	8	6	0.5	4	6
1	10	10	4	6	1	0.25	5	5	1	10	10	4	8	8	0.5	2	6
1	10	10	6	6	1	0.25	5	4	1	10	10	6	8	2	0.5	6	5
1	10	10	8	6	1	0.25	5	3	1	10	10	6	8	4	0.5	5	5
1	10	10	2	8	1	0.25	6	6	1	10	10	6	8	6	0.5	4	5
1	10	10	4	8	1	0.25	6	5	1	10	10	6	8	8	0.5	1	5
1	10	10	6	8	1	0.25	6	5	1	10	10	8	8	2	0.5	5	4
1	10	10	8	8	1	0.25	5	3	1	10	10	8	8	4	0.5	4	4
2	10	8	4	2	1	0.25	2	3	1	10	10	8	8	6	0.5	4	4
4	10	8	4	2	1	0.25	2	3	1	10	10	8	8	8	0.5	1	4
6	10	8	4	2	1	0.25	1	3	1	10	4	2	2	1	1	3	4
8	10	8	4	2	1	0.25	1	2	1	10	6	2	2	1	1	3	6
10	10	8	4	2	1	0.25	1	2	1	10	6	4	2	1	1	3	4
1	2	8	4	2	1	0.25	2	5	1	10	8	2	2	1	1	3	7
1	5	8	4	2	1	0.25	3	5	1	10	8	4	2	1	1	3	6
1	20	8	4	2	1	0.25	2	4	1	10	8	6	2	1	1	3	4
1	50	8	4	2	1	0.25	2	4	1	10	10	2	2	1	1	3	8
1	100	8	4	2	1	0.25	2	4	1	10	10	4	2	1	1	3	7
1	10	4	2	2	1	0.5	3	4	1	10	10	6	2	1	1	2	6
1	10	6	2	2	1	0.5	3	5	1	10	10	8	2	1	1	3	4
1	10	6	4	2	1	0.5	3	4	1	10	6	2	4	1	1	5	6
1	10	8	2	2	1	0.5	3	6	1	10	6	4	4	1	1	5	4
1	10	8	4	2	1	0.5	3	5	1	10	8	2	4	1	1	5	7
1	10	8	6	2	1	0.5	3	4	1	10	8	4	4	1	1	5	6
1	10	10	2	2	1	0.5	3	7	1	10	8	6	4	1	1	5	4
1	10	10	4	2	1	0.5	3	6	1	10	10	2	4	1	1	5	8
1	10	10	6	2	1	0.5	3	5	1	10	10	4	4	1	1	5	7
1	10	10	8	2	1	0.5	3	4	1	10	10	6	4	1	1	6	6
1	10	6	2	4	1	0.5	5	5	1	10	10	8	4	1	1	5	4
1	10	6	4	4	1	0.5	4	4	1	10	8	2	6	1	1	7	7
1	10	8	2	4	1	0.5	5	6	1	10	8	4	6	1	1	7	6
1	10	8	4	4	1	0.5	5	5	1	10	8	6	6	1	1	6	4
1	10	8	6	4	1	0.5	4	4	1	10	10	2	6	1	1	6	8
1	10	10	2	4	1	0.5	4	7	1	10	10	4	6	1	1	7	7
1	10	10	4	4	1	0.5	5	6	1	10	10	6	6	1	1	7	6
1	10	10	6	4	1	0.5	5	5	1	10	10	8	6	1	1	5	4
1	10	10	8	4	1	0.5	4	4	1	10	10	2	8	1	1	8	8
1	10	8	2	6	1	0.5	6	6	1	10	10	4	8	1	1	8	7
1	10	8	4	6	1	0.5	6	5	1	10	10	6	8	1	1	7	6
1	10	8	6	6	1	0.5	5	4	1	10	10	8	8	1	1	6	4
1	10	10	2	6	1	0.5	6	7	2	10	8	4	2	1	1	2	4
1	10	10	4	6	1	0.5	6	6	4	10	8	4	2	1	1	2	4
1	10	10	6	6	1	0.5	6	5	6	10	8	4	2	1	1	1	3
1	10	10	8	6	1	0.5	5	4	8	10	8	4	2	1	1	1	3
1	10	10	2	8	1	0.5	7	7	10	10	8	4	2	1	1	1	3
1	10	10	4	8	1	0.5	7	6	1	2	8	4	2	1	1	3	6
1	10	10	6	8	1	0.5	7	5	1	5	8	4	2	1	1	3	6
1	10	10	8	8	1	0.5	5	4	1	20	8	4	2	1	1	3	6
2	10	8	4	2	1	0.5	2	4	1	50	8	4	2	1	1	2	6
4	10	8	4	2	1	0.5	2	3	1	100	8	4	2	1	1	3	6
6	10	8	4	2	1	0.5	2	2	1	10	4	2	2	1	2	4	5
8	10	8	4	2	1	0.5	1	3	1	10	6	2	2	1	2	3	7
10	10	8	4	2	1	0.5	1	3	1	10	6	4	2	1	2	5	6
1	2	8	4	2	1	0.5	3	6	1	10	8	2	2	1	2	3	8
1	5	8	4	2	1	0.5	3	5	1	10	8	4	2	1	2	3	7
1	20	8	4	2	1	0.5	3	5	1	10	8	6	2	1	2	4	5
1	50	8	4	2	1	0.5	2	5	1	10	10	2	2	1	2	3	10
1	100	8	4	2	1	0.5	3	5	1	10	10	4	2	1	2	3	9
1	10	4	2	2	2	0.5	1	4	1	10	10	6	2	1	2	3	8
1	10	6	2	2	2	0.5	1	5	1	10	10	8	2	1	2	5	6
1	10	6	4	2	2	0.5	1	4	1	10	6	2	4	1	2	6	7
1	10	8	2	2	2	0.5	2	6	1	10	6	4	4	1	2	6	5
1	10	8	4	2	2	0.5	2	5	1	10	8	2	4	1	2	6	9
1	10	8	6	2	2	0.5	1	4	1	10	8	4	4	1	2	7	7
1	10	10	2	2	2	0.5	2	7	1	10	8	6	4	1	2	6	5
1	10	10	4	2	2	0.5	2	6	1	10	10	2	4	1	2	6	10
1	10	10	6	2	2	0.5	1	5	1	10	10	4	4	1	2	6	9
1	10	10	8	2	2	0.5	1	4	1	10	10	6	4	1	2	6	7
1	10	6	2	4	2	0.5	4	5	1	10	10	8	4	1	2	6	5
1	10	6	2	4	4	0.5	2	5	1	10	8	2	6	1	2	8	9
1	10	6	4	4	2	0.5	4	4	1	10	8	4	6	1	2	7	7
1	10	6	4	4	4	0.5	1	4	1	10	8	6	6	1	2	6	5
1	10	8	2	4	2	0.5	4	6	1	10	10	2	6	1	2	7	9
1	10	8	2	4	4	0.5	2	6	1	10	10	4	6	1	2	8	9
1	10	8	4	4	2	0.5	4	5	1	10	10	6	6	1	2	8	7
1	10	8	4	4	4	0.5	1	5	1	10	10	8	6	1	2	6	5
1	10	8	6	4	2	0.5	4	4	1	10	10	2	8	1	2	10	10
1	10	8	6	4	4	0.5	1	4	1	10	10	4	8	1	2	9	9
1	10	10	2	4	2	0.5	4	7	1	10	10	6	8	1	2	9	7
1	10	10	2	4	4	0.5	2	7	1	10	10	8	8	1	2	6	6
1	10	10	4	4	2	0.5	4	6	2	10	8	4	2	1	2	2	5
1	10	10	4	4	4	0.5	2	6	4	10	8	4	2	1	2	2	4
1	10	10	6	4	2	0.5	4	5	6	10	8	4	2	1	2	1	3
1	10	10	6	4	4	0.5	1	5	8	10	8	4	2	1	2	1	3
1	10	10	8	4	2	0.5	4	4	10	10	8	4	2	1	2	1	2
1	10	10	8	4	4	0.5	1	4	1	2	8	4	2	1	2	3	5
1	10	8	2	6	2	0.5	5	6	1	5	8	4	2	1	2	3	7
1	10	8	2	6	4	0.5	4	6	1	20	8	4	2	1	2	3	7
1	10	8	2	6	6	0.5	2	6	1	50	8	4	2	1	2	2	8
1	10	8	4	6	2	0.5	5	5	1	100	8	4	2	1	2	2	8
1	10	8	4	6	4	0.5	4	5									
